# Mainstreaming a gender perspective into food system policies for healthy diets in low- and middle-income countries: a policy landscape analysis

**DOI:** 10.1080/16549716.2025.2557651

**Published:** 2025-10-30

**Authors:** Mario Sibamenya Venance, Erica Reeve, Nestor Alokpaï, Elaine Q. Borazon, Samali Perera, Anne Marie Thow, Jody Harris

**Affiliations:** a Buhigwe District Council, Division of Health, Social Welfare and Nutrition Services, Kigoma, Tanzania; b Institute for Health Transformation, Global Centre for Preventive Health and Nutrition, School of Health and Social Development, Faculty of Health, Deakin University, Geelong, Australia; c School of Rural Sociology and Agricultural Extension, University of Agriculture, Porto-Novo, Benin; d International Graduate Program of Education and Human Development, College of Social Sciences, National Sun Yat-Sen University, Kaohsiung City, Taiwan; e Menzies Centre for Health Policy and Economics, School of Public Health, The University of Sydney, Sydney, Australia; f Institute of Development Studies, Brighton, UK; g Food Equity Centre, World Vegetable Centre, Bangkok, Thailand

**Keywords:** Gender, food systems, fruits and vegetables, healthy diets, low and middle-income countries

## Abstract

**Background:**

Globally, gender inequalities and inequities persist in the food system, with women lacking access to productive resources and decision-making roles. Policy can help address these issues, but the extent of gender consideration in policy in low- and middle-income countries remains unclear.

**Objective:**

The study aimed to document how governments are addressing gender issues in food systems for healthy diets and suggest ways to enhance gender responsiveness of policy with specific reference to fruit and vegetables.

**Methods:**

We used the Food Systems Framework and a gender and food systems analytical framework to analyze food systems policy documents relating to fruits and vegetables in Benin, the Philippines, Sri Lanka and Tanzania. Data were synthesized in a matrix to identify strengths and gaps in current policies to support vegetable production, distribution and consumption as part of healthy diets.

**Results:**

Although there was some diversity in approaches to gender in the policies in study countries, there were common findings across countries including the underrepresentation of women in policy-making processes, and limited integration of gender issues in food system policy that limits their influence on healthy diets. Very few policy documents explicitly draw on sex-disaggregated data despite its role in evidence-based policymaking

**Conclusion:**

Gender issues are noted in the food system policies of the study countries but not effectively acted upon: Women still face systemic discrimination in food systems. This gap highlights a key area for enhancing policy design and execution. Sex-disaggregated data are critical for evidence-based food system policymaking; however, very few of the policy documents examined in the study countries.

## Paper Context

- **Main findings**: While the study countries have ratified conventions and implemented policies promoting gender equality in the food system, women still face systemic discrimination within the food systems.
- **Added knowledge**: This study is the first independent policy landscape analysis of how governments are using policy to acknowledge and empower women to contribute to fruit and vegetable production and consumption for healthy diets across food systems.
- **Global health impact for policy and action**: Mainstreaming a gender perspective into food system policies helps to achieve global healthy diets. This is because gender disparity inhibits poverty reduction, health, education, and climate change.

## Background

Governments around the world have committed to reorienting food systems so that they better deliver across social, environmental and health outcomes [1]. This paper focuses on a major social issue: gender-based inequality and the inequities that shape the food system; and a critical health issue: how these inequities impact access to diverse diets rich in fruits and vegetables, in four low- and middle-income countries.

### Women’s roles are vital in food systems and nutrition security

Women play an important role in food systems in low- and middle-income countries (LMICs), which provide livelihoods for women as well as men [2,3], and feed us all. In developing countries, women produce 60–80% of food and are estimated to contribute half of world production [4]. Women have a crucial role in seed preservation and biodiversity by contributing to the adaptability of crops to changing climate conditions and pests [5,6]. In addition to primary food production, rural women have also played a role in value addition and processing [7], including converting raw food products into consumable and marketable goods for domestic and community use [8]. Many rural women possess an extensive understanding of local ecosystems and utilise this expertise in the management of land, actively encouraging soil fertility, water preservation, and biodiversity [8]. Women, who may work as farmers or business operators or entrepreneurs throughout the food system, have a significant impact on productivity and agriculture-led growth, however, their role in food production and household food security is often under-recognized [4,8].

Beyond their roles as producers and suppliers of food, women more often face food and nutrition insecurity due to lower-paying positions, smaller incomes, and less control over household finances than men [3]. Concerning consumption, women in most contexts have a crucial impact on determining food purchases and meal preparations, which directly influence the health and well-being of their family members [2,9]. Women more often struggle to afford nutritious diets and in many contexts are the ones who travel to markets, especially for perishable foods such as fruits and vegetables [3]. Fruits and vegetables are a vital part of a healthy diet, and men and women alike do not eat enough of them, on average [10,11]. Gender inequality and other intersectional social disadvantage contribute to poor diets, malnutrition, and poverty [12]. Empowering women has been shown in multiple contexts to be one of the basic prerequisites for cultivating healthy diets and reducing gender gaps in opportunity and achievement [13].

### The gender gap hinders women

Food systems are not doing enough to address the needs and rights of women. Women contribute strongly to production, processing, distribution, and consumption; however, the system maintains inequities that reinforce gender inequality [8]. Inequalities in food systems have been defined as ‘the observed differences in food security and nutrition (FSN) outcomes, or related food systems factors (such as access to food production resources), between individuals and groups (when disaggregated by social, economic and geographical position)’; while inequities have been defined as ‘the avoidable reasons why uneven distribution exists and why disadvantage accrues systematically, based on asymmetries in social position, discrimination and power’ [14]. To clarify, sex is a biological term that refers to the physical characteristics of a person, while gender is a social construct that refers to the roles and expectations placed on a person related to masculinity and femininity [15]. In this work, we look at men and women, but we acknowledge the range of sex and gender issues with which broader society is also grappling.

Gender-based discrimination hinders women’s ability to get suitable employment, access services, acquire productive resources, and participate in markets [3]. The gender gap in food systems hinders women’s access to financing and credit, and their average income is lower than men’s [16,17]. For instance, from 2011 to 2021, the disparity in financial inclusion between genders has consistently stood at 7% worldwide and 9% in developing countries [18]. In fruits and vegetable subsector, women have been found in multiple studies to be specifically disadvantaged in terms of inputs and assets in small-scale commercial vegetable production, and tend to play a supportive role to men’s dominant role in production, with more participation in non-farm activities such as selling and preparing vegetables for sale; while women’s employment in commercial vegetable production tends to be more insecure and less senior [19].

Gender parity and the economic empowerment of women are crucial for the development of inclusive food systems [20]. Understanding the complex factors that influence gender equity in food systems and the opportunities to develop targeted policies and programs that promote gender equality within the food system is crucial to an equitable food system transformation [21]. Because, if rural women had equal access to land, technology, financial services, education, and agricultural markets, 100–150 million hungry people could be fed [22]. This projection shows why gender inequality is not simply ‘a women’s issue’ but is important to ending global hunger.

### Policies are critical to addressing gender inequality in food systems

One important factor in levelling the gender playing field is the role of policy (the system of laws, regulations and documentary commitments to action adopted by governments) in addressing the social and economic issues faced by women in societies. Policies and regulations have the potential to either facilitate or hinder the advancement of women, including women’s access to core resources such as land or credit – or to erode their rights [20]

Gender ‘mainstreaming’ (ensuring that gender issues are considered in all policies) is one approach that allows policymakers to overcome existing gaps and disadvantages that prevent women from fully participating in the food system [23,24]. Policies that are responsive to gender are very important because women and men may have different needs, priorities, and stresses (in agriculture, health, and other sectors) that may lead to the unequal distribution of resources and services, which, in turn, can lead to gender inequalities [25]. Government attention to ensuring that agricultural and other food system policies address the different needs, roles, and capacities of women and men, has the potential to make them more inclusive and effective [26,27].

For example, in Ethiopia, children’s calorie consumption and body mass index enhanced in regions where mothers possessed more secure land rights. Moreover, the same study indicated that enhanced access to land for women resulted in a 36% reduction in household food insecurity. Research in Nepal indicates that children whose mothers have access to land were 33% less likely to experience malnutrition. In Rwanda, studies observed improved child nutritional outcomes in families where women had control over agricultural decisions [28].

## Methods

This study is a part of a broader project that aims to increase fruit and vegetable intake in LMICs (Benin, Sri Lanka, the Philippines, and Tanzania), while also empowering women and youth and making fruit and vegetable sector jobs safer, more accessible, and profitable [29]. The study’s aim was to assess how governments are using policy to acknowledge and empower women to contribute to fruit and vegetable production and consumption across food systems, and propose strategies to improve gender responsiveness at the policy level, specifically in relation to fruit and vegetables.

### Study frameworks

**Figure 1.**
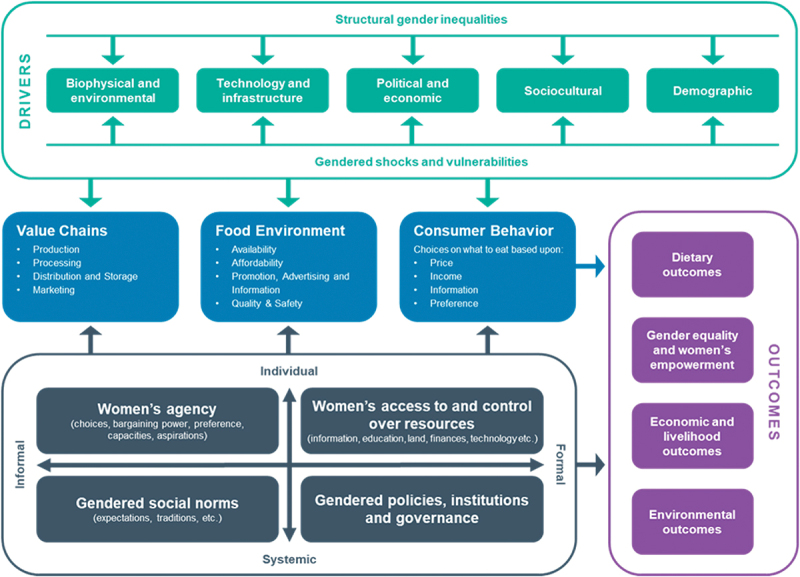
Gender and food systems framework. Source: (3) Njuki J, Eissler S, Malapit H, Meinzen-Dick R, Bryan E, Quisumbing A. A review of evidence on gender equality, women’s empowerment, and food systems. Global Food Security, 33, 100622. 2022.

The major framework used to frame the study and analyze emerging findings was the Gender and Food Systems Framework [20] (Figure 1). This framework is built on an accepted conceptualization of food systems comprising supply chains, food environments, and consumer behavior shaped by biophysical, political, economic, and socio-cultural drivers [30], overlaid with reference to essential terminology and definitions related to women’s empowerment, women’s economic empowerment, and gender-transformative strategies [20]. We employed a gender and food systems analytical framework as it emphasises aspects that encompass both formal and informal structures, individual and systemic transformations, with an aim to enhance women’s agency, improve access to and rights over resources, including land, alter detrimental gender and social norms, and eliminate policy, legal, and institutional impediments [31]. The key policy sectors that correspond to the framework were: Agriculture, Fisheries, Industry/Commerce, Infrastructure, Trade, Finance, Environment, Health, and Gender (the names of the responsible ministries differ across country contexts).

### Case study countries

Four target countries were chosen based on their low fruit and vegetable consumption and the potential for significant impact within a three-year project timeframe. These countries, Benin, Tanzania, Sri Lanka, and the Philippines, also have existing partnerships and a clear governmental interest in transforming food systems and mitigating malnutrition [29]. All four countries have notable agricultural production, particularly in the cultivation of fruits and vegetables, with very different socio-cultural contexts. It has been noted that all of them experience various gender inequalities and inequities that impact not only the production and consumption of fruits and vegetables but also health, education, economy and politics [32].

In 2006, the World Economic Forum launched the Global Gender Gap Index to track gender parity and evaluate gender differences in economic opportunities, education, health, and political leadership [33]. The gender gap is defined as the ‘difference between women and men as reflected in social, political, intellectual, cultural, or economic attainments or attitudes’ and therefore covers aspects of inequality and inequity [33]. The index of gender inequality is the central metric used to measure gender equity at the national level and is calculated based on factors such as reproductive health, education, political participation, and labour force participation [34]. According to a range of indices, none of the focus countries has been able to guarantee gender parity. All of our selected countries are in the bottom half of gender equity rankings by most metrics (Table 1). Women and girls in the research countries have been put at a disadvantage due to inequality in political representation, impediments in the labour market, disparities in skill development, and gendered societal norms and practices [35]. These four countries represent a range of contexts in terms of fruit and vegetable consumption, women’s rights, and food systems, across two continents.

**Table 1.** Study countries’ position on global gender indices.

Index	Countries (n)	Value and rank			
		Benin	Sri Lanka	Philippines	Tanzania
The Global Gender Gap Index 2023 rankings	146	Value:0.616 Rank: 138^th^	Value:0.663 Rank: 115^th^	Value:0.791 Rank: 16^th^	Value:0.740 Rank: 48^th^
Human Development Index (HDI) 2022^a^	193	Value:0.504 Rank: 173^rd^	0.780 Rank: 78^th^	Value: 0.710 Rank: 113^rd^	Value 0.532 Rank: 167^th^
Gender Development Index (GDI) 2022^a^	191	Value: 0.880 Rank: 166^th^	Value: 0.949 Rank: 73^rd^	Value: 0.990 Rank: 116^th^	Value: 0.943 Rank: 160^th^
Gender Inequality Index (GII) 2022^a^	193	Value: 0.649 Rank: 160^th^	Value 0.376 Rank: 90^th^	Value:0.388 Rank: 92^th^	Value:0.513 Rank 131^st^
Global Gender Gap Index (GGGI) 2023^b^	146	Value: 0.616 Rank: 138^th^	Value: 0.663 Rank:115^th^	Value: 0.791 Rank: 16^th^	Value: 0.740 Rank: 48^th^
Women’s Economic Opportunity Index (WEOI) 2012^c^	128	Value: 40.8 Rank: 101^th^	Value: 47.6 Rank: 84^th^	Value: 50.3 Rank: 74^th^	Value: 45.4 Rank: 95^th^

^a^(UNDP, 2022).

^b^(World Economic Forum, 2023).

^c^(Economic Intelligence Unit, 2012).

### Inclusion and exclusion criteria

We defined policy as the way governments translate their vision and objectives into actions in the real world. We selected policy documents that met the following inclusion criteria (Table 2): 1) The policy should either be regulations, policy documents, strategies, action plans, or guidance documents that are published in English, French, or Swahili, and specifically relating to our four focus countries. 2) The relevant policy documents across sectors must relate to fruits and vegetables in food systems.

**Table 2.** A summary of the types of policy documents reviewed.

Type of document	Number of policy documents reviewed				
	Benin	Philippines	Sri Lanka	Tanzania	Total
National Development plan	2	1	0	1	5
Policy	1	0	17	13	31
Strategy/Action plan	11	4	10	6	31
Guideline/Framework	0	0	2	4	6
Programme	1	1	0	2	4
Act/Order/Decrees/Regulation	1	47	6	4	58
**Total**	**16**	**53**	**36**	**30**	**135**

### Data collection

A search for current national policy documents was conducted between July and October 2022 in each country. All policies relating to specific aspects of food systems were systematically sought on government websites and websites of international agencies collating food system policies, and sectoral experts were consulted about gaps in documents found and additional documents included if they met inclusion criteria. A total of 135 policy documents were included across the four countries (Table 2).

Beyond national policies, an extensive search was conducted on the UN Women website for reports from ‘grey literature’ pertaining to key international legal frameworks and regional treaties signed or ratified by study countries and how these have been translated into Constitutions and domestic policies (Table 3).

**Table 3.** Ratification of international gender equality agreements in the study countries.

International agreements	Ratification and Year							
	Benin		Philippines		Sri Lanka		Tanzania	
Convention on the Elimination of All Forms of Discrimination Against Women (CEDAW)	Yes	1992	Yes	1981	Yes	1981	Yes	1985
Convention on the Political Rights of Women (CPRW)	No	–	Yes	1957	No	–	Yes	1975
Optional Protocol to the Convention on the Elimination of all forms of Discrimination Against Women (OP-CEDAW)	Yes	2018	Yes	2003	Yes	2002	Yes	2006
Beijing Platform for Action (BPA)	Yes	1996	Yes	1995	Yes	1995	Yes	1995
International covenant on economic, social and cultural rights	Yes	1992	Yes	1974	Yes	1980	No	–
Gender Equality and the Sustainable Development Goals	Yes	2015	Yes	2015	Yes	2015	Yes	2015

### Data extraction and analysis

A comprehensive keyword search was employed within each policy document to identify places where gender issues were discussed. The search terms employed in our study were informed by the Gender and Food System Framework and included: gender, women, female, equality, and equity. We constructed an excel matrix where extracted text was entered for the broader project, across areas including consumer behavior, Safe and Sustainable Production Systems, Post-harvest and inclusive markets, Food environments, policy framing and beliefs, governance structures, budget allocations, implementation mechanisms, and specific mentions of gender and equity issues. For this study, the matrix was examined to identify issues explicitly and implicitly showing how gender is addressed through policy approaches. We summarized the key policy findings related to gender mainstreaming based on the gender aspects described in the Gender and Food System Framework.

## Results

### Gendered policies, institutions and governance of study countries

All four governments of the case study countries have formally signed on to global gender initiatives (Table 3). For example, the Sustainable Development Goals that all the study countries have signed have informed the development of specific gender-focused policies and programs in the study countries, as well as set the scene for mainstreaming of gender in other food system policies. However, not all countries have signed up to all relevant agreements that would support a gender-responsive food system. For instance, Benin and Sri Lanka have not yet ratified the Convention on the Political Rights of Women (CPRW).

However, gender issues are integrated into national policies in all four study countries, reflecting commitments undertaken to promote equality and women’s empowerment. In addition, all four constitutions of the studied countries guarantee equality for all individuals irrespective of their gender. In Benin, policies focus on enhancing women’s access to education, economic opportunities, and participation in decision-making, particularly in rural areas. Sri Lanka puts a strong emphasis on gender equality in education and health, with policies targeting women’s entrepreneurship and protection against gender-based violence. The Philippines has advanced legal frameworks, such as the Magna Carta of Women, promoting gender equality in governance, labor, and social services. Tanzania integrates gender considerations into agriculture, health, and education policies, recognizing women’s roles in rural economies and their vulnerabilities to poverty and health disparities (Table 4).

**Table 4.** Highlights from policy documents aimed at promoting the food system and women’s economic empowerment.

Country	Policy	Year	Policy highlights	Relevance to A gender and food systems analytical framework*
Benin	Constitution of the People’s Republic of Benin	1990	Is obligated to ensure that all individuals are treated equally under the law, regardless of their origin, race, gender, religion, political viewpoint, or socioeconomic status	Promote equal access to productive resources that could make women to engage freely in food system.
	National Gender promotion Policy of Benin	2008–2025	Emphasize on the equality and equity and encouraging the participation of women and men in decision-making, access to and control of resources for sustainable development Emphasizes the use of sex-disaggregated data in decision-making	Women are actively involved in various stages of food production, processing, and marketing.
	National Policy for the Promotion of Women in Agriculture	2001	It aims to enhance rural people’ living conditions by giving women and men equal opportunity to participate in Benin’s development. The strategic objectives are among others: 1) Implement measures to make equality and equity between men and women in access to education, literacy and health care structures decisions in all spheres (individual, family, community, national and international); 2) Strengthen the institutionalization of gender at all levels, as well as the effective application of national and international conventions and texts favourable to equality and fairness between men and women; 3) Strengthen civil society engagement and awareness women and men for the promotion of gender while ensuring good involvement men in the process; 4) Ensuring the empowerment of women and better consideration of gender in the Communal Development Plans (PDC); 5) Reduce women’s monetary poverty and provide them with equitable access and control to resources	Women play a crucial role in the food system, particularly in rural areas where agriculture is a primary livelihood activity
	Decree no. 2009–728 on December 31, 2009, reviewed by the decree N’2021–391 of July 21, 2021	2009 and 2021	The mission of the National Institute of Women is to work for the promotion of women at the political, economic, social, legal and cultural levels in both the public and private spheres and to fight against all forms of discrimination and violence towards women.	Help to educate women on taking initiative for food production and healthy diets
	National Policy on Girls’ Education and Training	2007	To close the gender gaps in education and training in Benin	Education for women and girls not only grants them access to improved employment prospects inside food systems, but also empowers them to actively pursue the chances of their choice, both within and beyond food systems)
	Land and domain code in Benin Republic (2013 and revised in 2017)	2013 and reviewed in 2017	Ensure equitable access to land for all actors, natural persons and legal entities under public and private law Ensure respect for the gender approach in access to land.	Promote equity in access to land for both women and other vulnerable people for agricultural production
	National Plan for Agricultural Investment and Food and Nutrition Security (PNIASAN)	2017–2025	The PNIASAN approach advocates specific support for women and young people to enable them to have better access to productive resources and markets.● Strengthen education and vocational training programs for rural women,● promote the development and transfer of appropriate technologies for women, ● develop information and communication in favor of rural women for social change and ● strengthen the organizational capacities of rural women and their participation in peasant organizations.	Advocates specific support for women to enable them to have better access to productive resources and markets to agriculture produce
	National Fruit Tree Development Strategy (SNDAF-2020–2025) with particular emphasis on the orange, mango and papaya sub-sectors	2020–2025	It is concerned especially with doubling, by 2030, the agricultural productivity and the revenue of small farmers and especially of women Taking actions towards awareness, outreach, education, guidance and support initiatives for agro-entrepreneurs, particularly to attract interest among women among other groups The sub-program focuses on raising awareness about the opportunities available to women who are takin g action.	Investing in education, information and financial services, employment opportunities, and empowering women can improve food security and nutrition outcomes, as well as amplify their role in strengthening the food system’s resilience.
	Communal Development Plan of Cotonou	2018–2022	Improve food security, nutrition, and access to basic socio-community services (such as education, health, and water), as well as gender equality. Facilitate women’s access to microcredits, promote youth entrepreneurship, and boost decentralized cooperation.	The gender gap in food systems hinders women’s access to financing and credit, and their average income is lower than men’s. Increase women access to credit could help them to excel into value chain.
	National Gender Promotion Policy (PNPG)	2008–2025	Focused on improving access to education for girls Improving access to sexual and reproductive health services. Combatting Gender-Based Violence	Initiatives aimed at promoting gender equality and empowering women, contribute to society and economic growth.
	National School Feeding Policy	2014–2025	Identify all schools in vulnerable areas that can benefit from canteens on the basis of clearly defined selection criteria	Promote access to rich diets for school children in rural and vulnerable areas
	The Holistic Social Protection Policy (PHPS)	2014–2025	The Government will strengthen preventive measures, support and promotion of social groups in a situation of high vulnerability to the risk of abuse, violence, exploitation, discrimination or exclusion. These groups include: ● children living outside a family setting (street children, children abandoned or delivered to orphanages, children in detention), child victims of trafficking, children involved in the worst forms of child labor and child victim’s violence and abuse, among other categories of high-risk children; ● women who are victims of violence or abandoned without support by their spouses, and girls who are victims of harmful practices such as excision and early marriage; ● elderly people who are abandoned, living in isolation, victims of abuse or deprived; ● people with disabilities living in a situation of social exclusion (especially from school and jobs), stigmatized, and without the support in terms of medication, equipment, rehabilitation and reintegration that they need	Help to promote access to required food to vulnerable people through social nets facilities
	The Health Sector Policy for Nutrition (PSSN)	2016–2025	This health sector policy for nutrition is focused on mothers, infants, children and adolescents, Some of the activities are targeting vulnerable people such as: The monitoring of the nutritional status of women and children The Nutritional support for people with HIV/AIDS and/or tuberculosis	Contribute to improving required food access for women and children and people affected by some specific illnesses
	National program for the development of the vegetable crops sub-sector	2017–2021	To sustainably improve the production, productivity and competitiveness of market gardening products in Benin	(i) promoting rural women’s access to land in a secure manner, (ii) making credit more accessible to rural women, (iii) improving rural women’s access to agricultural inputs, (iv) promoting the development of agricultural production for the benefit of rural women and (v) promoting rural women’s access to remunerative employment (vi) Strengthening Women’s Organizing Capacities and their participation in farmers’ organizations
Sri Lanka	Constitution of the Democratic Socialist Republic of Sri Lanka	1978	Chapter 3, Article 12 ensures the protection of basic rights and prohibits discrimination based on gender.	Promote equal access to productive resources that could make women to engage freely in food system.
	Policy Framework and National Plan of Action to Address Sexual and Gender -Based Violence	2016	Adopts a rights-based strategy to tackle gender-based violence (GBV) with the aim of fostering a life free from violence for women and children.	Addressing Gender-based violence has an overwhelming impact on agricultural productivity, food and nutrition security
	Draft National Policy on Women	2019	The National Women’s Policy aims to standardize and steer the development of laws, policies, programs, and mechanisms. Additionally, the policy seeks to provide equal rights and opportunities for women in government, the workplace, community, family, and civic spaces.	Provision of equal rights and opportunities for women in government, the workplace, community, family, and civic spaces.
	National Strategic Plan on Adolescent and Youth Health	2018–2025	Eliminate gender disparities in education and ensure equal access toall levels of education and vocational training for children in vulnerablesituations Adopt and strengthen sound policies and enforceable legislations on thepromotion of gender equality and the empowerment of girls at all levels- page Prevention and response to sexual and other forms of gender-based violence	Education for women and girls enhances employment opportunities within food systems and empowers them to pursue their desired opportunities both within and beyond food systems.
	National Agriculure Policy (Draft under the cabinet)	2021–2030	The focus is on creating suitable technologies to retain youth, boost women’s sector participation, and ensure equal opportunities for women in Agriculture.	The use technology could reduce women work load and make them to involve more in food system
	National Agriculture Research Policy and Strategy	2018–2027	Strengthening local service providers’ capacity to support farmers, particularly farm women, farmer organizations, rural communities, and government agencies’ human capital.	farmer organizations increase voice for changes in the food system
	National Agriculture Policy	2007	Promote home gardening and urban agriculture to enhance household nutrition and income. Promote women’s participation in home gardening	Home gardening promote urban agriculture
	National Policy for decent work in Sri Lanka	2006	To achieve a future of peace, prosperity, and improved quality of life for all citizens, (women and men) free from poverty and deprivation.	Addressing poverty goes hand in hand with improved agriculture system especially in rural areas.
Philippines	The 1987 Constitution of The Republic of The Philippines	1987	Affirm women’s rights to own property, contract employment, and credit without husbands’ consent	Granting women, the right to own land, this will enable women to increase agriculture productions.
	Philippine Development Plan for Women	1989–1992	Fighting against gender discrimination and ensuring women’s equal access to development resources.	Addressing Gender-based violence has an overwhelming impact on agricultural productivity, food and nutrition security
	Women in Development and Nation Building Act (R7192)		i) Guarantees women and men equal rights, opportunity, and legal equality; ii) equal access to government and private agricultural loan programmes; iii) Rural women are also encouraged to have equal access to resources and training.	Giving both men and women equal access to agricultural loan and training
	Philippine Plan for Gender-Responsive Development,	1995–2025	i)The government promoted mainstreaming GAD in strategies, policies, and programmes for implementation across government sectors; ii) It recognised that GAD Focal Points were essential to institutionalising GAD.	Promotion of gender mainstreaming, which including women’s and men’s concerns and experiences in the design, implementation, monitoring, and evaluation of policies, programmes, and projects in all social, political, civil, and economic sectors to benefit both genders.
	The Magna Carta of Women	2009	Aims to improve gender-responsive governance by encouraging women’s active involvement in programme creation and policy-making at all levels and in all sectors.	The promotion of gender-responsive governance involves actively supporting women’s participation in the establishment of programmes and the development of policies across all levels and sectors.
	Philippine Development Plan	2017–2022	Create more jobs and economic opportunities for the youth and women by providing access to productive employment Emphasises the importance of sex-disaggregated data in monitoring and evaluating policies and programmes’ effects on different gender groups	Education for women and girls enhances employment opportunities within food systems and empowers them to pursue their desired opportunities both within and beyond food systems.
	RA 8435: Agriculture and Fisheries Modernization Act (AFMA) of 1997	1997– 2003	To implement special training projects for women for absorption in the basic needs and rural industrialization programs	
			To alleviate poverty and promote vigorous growth in the countryside through access to credit by small farmers, fisherfolk, particularly the women involved in the production, processing and trading of agriculture and fisheries products and the small and medium scale enterprises (SMEs) and industries engaged in agriculture and fisheries.	Addressing poverty goes hand in hand with improved agriculture system especially in rural areas.
	RA 10,068-Organic Agriculture Act of 2010/RA 11,511	2010	Research, development and extension of appropriate, sustainable environment and gender-friendly organic agriculture	Extension services to women are critical as far as food system is concern
	MSME Development Plan 2017–2022	2004–2022	Enhance labor capacities through human resource development and gender sensitivity programs for MSMEs	MSMEs are important for strengthening food system
	RA 9710: An Act providing for the magna carta of women	2009	The policy aims to uphold women’s right to participate in policy formulation, planning, organization, implementation, management, monitoring, and evaluation, while supporting policies, research, technology, training programs, and support services.	Gender-responsive agriculture can improve sustainability and equity through policy measures
	PO-2011-041 R: an ordinance institutionalizing organic agriculture in bukidnon, providing funds therefor and for other purposes	2011	Acknowledges women’s role in organic agriculture and will ensure gender-fair development, supporting women like child-bearing girls, tender-age girls, and elderly farm workers.	
Tanzania	The Constitution of The United Republic of Tanzania	1977	Acknowledges the principle of equal property ownership rights for all its residents.	Acknowledgement of the principle of equal property ownership rights for both men and women.
	National Strategy for Gender Development	2005	To attain gender equality and equity in Tanzania, as outlined in the National Constitution and the Women and Gender Development Policy.	Promote equal access to productive resources that could make women to engage freely in food system.
	National Anti – Female Genital Mutilation (FGM) Strategy and Implementation Plan	2020/21–2024/215	Preventing the practice of female genital mutilation (FGM) on girls and women, and to address the needs of survivors, it is necessary to implement retributive justice against the perpetrators. This can be achieved by thorough coordination across many sectors at all levels	Addressing Gender-based violence has an overwhelming impact on agricultural productivity, food and nutrition security
	National Plan of Action for the Prevention and Eradication of Violence Against Women and Children	2017/18–2021/22	Eradicate the occurrence of violence against women and children in Tanzania and enhance their well-being.	Addressing Gender-based violence has an overwhelming impact on agricultural productivity, food and nutrition security
	Tanzania’s Development Vision (TDV 2025)	1995–2025	Redressing gender imbalances in all economic activities by the year 2025	Addressing Gender-based violence has an overwhelming impact on agricultural productivity, food and nutrition security
	Food and Nutrition Policy	1992	Encouraging the use of better technology to farmers to reduces women workload	The use technology could reduce women work load and make them to involve more in food system
	National Health Policy	2007	Improving the integration of women’s empowerment and gender equality into health care. Strengthening intersectoral strategies to combat violence against children (VAC) and gender-based violence (GBV)	Improving women health have a direct impact on increasing performance on agricultural productivity
	National Multi-sectoral Nutrition Action Plan	2021–2026	Women, men, children, and adolescents in Tanzania experience improved nutrition, leading to enhanced health and increased productivity in their life.	Improving women health have a direct impact on increasing performance on agricultural productivity
	National Agriculture Policy	2013	Equal access to land between men and men Promotion of participatory approaches and gender aspects in the provision of extension service	Granting women, the right to own land, this will enable women to increase agriculture productions.
	National Horticulture Development Strategy and Action Plan	2021–2031	To improve the abilities of women and young people, provide them with financial services, and include them in the horticultural sector. To enhance women and youth skills, access to financial services and participation in the horticulture industry”	Women face disadvantages in small-scale commercial vegetable production due to financial inclusion disparities in developing countries.
	The National Irrigation Policy	2010	Insists on active and effective participation of both women and men in irrigation development	Irrigation water are necessary for agricultural productivity
	National Trade Policy	2003	Increasing the equality of access to economic assets, particularly land, infrastructure, financing, education, and skills, to include women in the development of commerce	If rural women had equal access to land, technology, financial services, education, and agricultural markets, agricultural productivity will increase

*A gender and food systems analytical framework are framed on four essential domains that encompass both formal and informal sectors, as well as individual and systemic transformations. Emphasizing on enhancing women’s agency, increasing access to and rights over resources such as land, altering detrimental gender and social norms, and eliminating policy, legal, and institutional obstacles.

## Women’s access to and control over resources

Food system policy documents in Benin, Tanzania, Sri Lanka and the Philippines included policy actions that aim to enhance the equitable distribution of economic resources and opportunities, with a specific focus on land, infrastructure, financing, education, and skills, to incorporate women in the advancement of commercial activities. For instance, the National Agriculture Research Policy and Strategy in Sri Lanka aimed at strengthening the capacity of local service providers to support farmers specially farm women (in land ownership, gender sensitizing technology and ownership to capital), farmer organizations and rural communities, as well as human capital of government agencies. In Tanzania, the Food and Nutrition Policy emphasizes the use of better technology to farmers to reduce women’s workload, for instance (Table 4).

Although the majority of policy documents identify gender as a cross-cutting issue, they do not have concrete measures to ensure the involvement of women in policy design and implementation. In other cases, policy documents mention the role of women in food production and nutrition but do not mention structural issues such as their limited access to land, finance, and leadership positions in governance structures.

## Norms and power dynamics of gender

Across all four countries, power dynamics and gender norms shape food system policy through the identification of who participates in decision-making and who benefits from policy interventions. Policies and legal frameworks tend to acknowledge gender equality and strongly held socio-cultural norms. Tanzania, the National Agriculture Policy (2013), identifies gender integration due to customary practices that restrict women’s mobility as well as their access to land, thereby discouraging their active participation in the food system. Gender norms in Benin are influential in shaping and determining decision-making in food systems. For instance, the National Policy for Gender Promotion (2009) aims to enhance women’s access to resources and participation in decision-making within the agricultural sector. The Philippines has been very progressive in integrating gender equality in its food system policies, particularly in agrarian reform and fisheries management. For example, the Magna Carta of Women (2009) enforces the incorporation of gender dimensions in agricultural and fisheries policies. Gendered power relations in Sri Lanka are shaped by both traditional norms and policy interventions. Policy Example, The National Policy on Women (2014) seeks to promote gender equality, most visibly in work and entrepreneurship.

## Women’s agency and leadership in policy processes

Policy documents in all study countries except Sri Lanka include policy actions that emphasize the need for women’s empowerment in decision-making. Policy provisions aim to empower women to facilitate their full and equitable participation alongside men in decision-making processes across food systems. For instance, Tanzania’s National Gender Policy supports gender equality by advocating for equal participation of women and men in economic, social, and political activities, including agriculture and food systems. Although policy documents in all four countries acknowledge the importance of women’s roles in food security and nutrition, they do not generally incorporate specific strategies for strengthening women’s voice in decision-making for policy development.

## Gender-sensitive data for programming

Our findings show that a large proportion of food systems policies in all four countries consider gender to some extent – even in policies that are not explicitly gender policies. More than half (59%) of the reviewed policies at least mention any gender-related issues, while 37% of documents explicitly consider gender in the policy statements with Tanzania being the leading one with 77% (Table 5). In Benin and Tanzania, gender issues were featured in their national development plan.

**Table 5.** Instances of gender considerations in policy.

Gender related questions	Pattern of response									
	Benin (*N* = 16)		Philippines (*N* = 53)		Sri Lanka (*N* = 36)		Tanzania (*N* = 30)		ALL (*N* = 135)	
	YES	NO	YES	NO	YES	NO	YES	NO	YES	NO
	N (%)	N (%)	N (%)	N (%)	N (%)	N (%)	N (%)	N (%)	N (%)	N (%)
Was gender mentioned in national development plan	2 100%	0 0%	1 100%	0 0%	0 0%	1 100%	1 100%	0 0%	4 80%	1 20%
Documents mentioning any gender related issue	6 38%	10 62%	11 21%	42 79%	14 39%	22 61%	24 80%	6 20%	55 41%	80 59%
Documents explicitly considering gender in policy statements	7 44%	9 56%	8 15%	45 85%	14 39%	22 61%	23 77%	7 23%	52 37%	83 62%
Documents that explicitly considering sex- disaggregated data	3 19%	13 81%	2 4%	51 96%	4 11%	32 89%	3 10%	27 90%	12 9%	123 91%

A further instance of gender considered in some policies is gender-disaggregated data – though only a few policies (9%) mention this (Table 5). In Tanzania, for example, one of the Policy Statement in the National Strategy for Gender Development, is to ‘ensure availability of gender disaggregated data and provision of guidelines that will enforce compliance to inclusion of gender/sex disaggregated data by actors at all levels’. The National Gender Policy of Benin emphasizes the use of sex-disaggregated data in decision-making. The policy recognizes that gender equality programmes must take into account women’s and men’s unique needs and experiences. The Philippines Development Plan emphasizes the importance of sex-disaggregated data in monitoring and evaluating policies and programmes’ effects on different gender groups. The Sri Lankan ‘National Policy on Social Integration’ promotes social inclusion and gender equality by collecting and using sex-disaggregated data.

## Discussion

The 2030 Agenda for Sustainable Development calls for greater gender mainstreaming [36]. Addressing gender inequality in food systems is a critical part of achieving this, and requires a multifaceted strategy. Policymakers can promote inclusivity and equity in food system policies by considering the unique needs and goals of women, through their active participation. However, at the moment, policy action to address gender equality in food systems is patchy [20]. Our study reveals that Benin, the Philippines, Sri Lanka, and Tanzania have diverse food systems policies that consider gender in diverse ways – but with multiple opportunities for strengthening.

## Mainstreaming gender in food system policy

Gender mainstreaming into food system policies in the study countries and beyond is required for equitable and sustainable health diet. Despite regional and international commitments to gender equality, gender mainstreaming in policy cycles in the study countries is limited to very few policies and faced by significant structural and institutional barriers. There is a lack of clear guidelines for incorporating gender equality into agriculture, specifically in the fruit and vegetable sector in the study countries. This absence of guidelines hinders the ability to promote the inclusion of women in agriculture, particularly in the fruit and vegetable sectors. Having guidelines for gender mainstreaming in the Ministry responsible for Agriculture would be a useful tool to ensure that gender-responsive actions are taken into account when implementing agricultural programs, strategies, and activities [37].

Gender mainstreaming in food system policies has shown significant improvements in dietary outcomes for women through various targeted interventions in various countries. Evidence from Rwanda, Nepal, Brazil, Bangladesh, Vietnam and Ethiopia demonstrates the many and varied possibilities to consider gender in policy, and positive impacts, especially in terms of how they might affect healthy diets within families [38]. These countries have implemented policies and programs aimed at raising the status of women in agriculture including land rights reforms, access to credit, and the creation of training platforms for women [39].

In Nepal, Women’s empowerment in agriculture, and particularly increased control over production decision-making and time allocation, was associated with increased maternal dietary diversity and child growth indicators, such as weight-for-age z-scores [40]. In Bangladesh, combined interventions on agriculture, gender, and nutrition were more effective compared to sectoral interventions [41]. In Brazil, the Fome Zero (Zero Hunger) policy framework has reduced food insecurity and malnutrition; specific gender policies increased access for women to finance and agricultural resources, translating into better family diets [42]. These multisectoral programs not only enhanced agricultural practices and women’s empowerment but also resulted in better household diet quality and greater child dietary diversity [41].

## Women’s access to and control over resources

One important strategy is to increase women’s access to productive resources such as land, credit and agricultural inputs. The findings from the policy documents show that some policies that emphasize gendered access to and control over resources do exist. However, evidence from studies shows that women are still gender discriminated in the food systems of the study countries. For instance, in Benin, women face significant barriers in obtaining land, despite the presence of Law No. 2013 of August 14 which addresses land ownership. Only 13% of women possess land, and a meagre 6% of them have secure land tenure rights [43]. Only 16% of all owned land in Sri Lanka belongs to women, and this limits their access to different agricultural assets and benefits such as subsidies, credit or irrigation water [37]. In Tanzania, the majority of women in the agriculture sector (67%) still face significant disadvantages in accessing productive inputs like land, labour, fertilizer, improved seeds, labour-saving technology, and finance [44].

The importance of women’s access to productive resources, including land, has been recognized in recent years. Research in Zambia and Nigeria showed that households headed by females usually have less dietary diversity than households headed by males probably because they are limited in their access to resources and their participation in subsidy programmes [45]. Expanding women’s access to these resources can close the existing gender gap in dietary diversity and enhance overall household nutrition in the study countries. In addition, education and agricultural extension services tailored to the specific needs of women could increase their ability to utilize these resources more effectively [45,46].

## Women’s agency and leadership in policy processes

Women’s leadership in policy processes is critical in achieving gender mainstreaming and improving social and economic outcomes. Women’s leadership in climate-resilient agrifood systems is increasingly recognized as being necessary to reduce the impact of climate change and to foster sustainable agriculture [47]. In the study countries, women’s agency and leadership in policy processes vary across the study countries, influenced by patriarchal norms and insufficient representation in political institutions. The Philippines and Tanzania had more policy documents that demonstrated the roles of women in decision making and leadership. They have also ratified the Convention on the Political Rights of Women. It is worth noting that the representation of women in the parliaments of Sri Lanka (5.4%) and Benin (8.4%) is significantly lower compared to Philippines (28%) and Tanzania (36.7%) [48,49].

Recent studies in Uganda and Bangladesh found that women’s empowerment, particularly regarding financial- and agricultural-related decision-making, is strongly linked to improved household food security and dietary diversity [50,51]. That is, policies that foster women’s leadership and agency, through gender-sensitive agricultural programs for example, will contribute to a better environment for nutrition. The policies and programmes that combine livelihood support with behavioral change communication have, however, evidenced greater effectiveness in sustaining improvements in dietary quality and food security [52,53]. However, gendered roles and responsibilities, particularly in unpaid care work, further limit women’s ability to engage in policy processes [54]. These challenges are compounded by the lack of gender-disaggregated data, which hinders the effective monitoring and evaluation of gender-responsive policies [55].

## Gender-sensitive data for programing

Addressing gender inequalities in the food system is a global challenge that requires evidence-based policy decisions towards equality [56]. The UN Agenda 2030 Sustainable Development Goals emphasize the importance of gender indicators in food system to monitor gender equality [36]. Collecting gender-disaggregated data is crucial for developing effective food system policies [56]. While there is now a growing emphasis on considering sex-disaggregated data in food systems policies globally [57], there are still gaps in the study countries. Only 9% of the reviewed policy documents explicitly considered sex-disaggregated data. These findings are similar to numerous studies which show that many agricultural and food system databases lack sex-disaggregated data, rendering the unique challenges and opportunities faced by women in agriculture and food-related sectors (16, 33 [58], 60–62).

## Norms and power dynamics of gender

Gender mainstreaming can facilitate changes in social norms and gender equality. A recent study in Ghana proved that integrating gender into agricultural policies caused a change from gender-blind to more inclusive approaches; however, much work needs to be done in putting more emphasis on gender equity [59]. The other study in Tanzania showed that efforts towards adopting gender-responsive agriculture have drawn even more attention to the need for dismantling rigid structures and gender norms if transformative change is to be achieved [60].

Social and cultural norms create and reinforce the ways in which women and men are able to participate in food systems, gain access to opportunities and resources [61–63]. They can conventionally deprive women of resources and opportunities, thereby also contributing to upholding inequalities in food systems [31,46]. Those policies that counter such norms can be instrumental in adjusting existing power relations leading to the establishment of food systems that are more equal.

Policies should also reflect the intersection of gender with other determinants: education, marital status, location, and so forth. Evidence from Tanzania and India suggests that individual contexts influence whether women have power or not; thus, initiatives against this need to be specific to the context. This means targeted interventions that would look into how urban women face challenges differently from rural women and how that could be remedied for equal resource placement and access [60,64].

A gender-sensitive approach to food system policies opens the door to strengthening nutrition and health benefits by empowering women and girls. Such policies could begin to address access to resources by recognizing decision-making power and gender norms in specific contextual factors, thus creating a food system that is more equitable and resilient for an entire community. Evidence from diverse contexts underscores the transformational possibilities of women’s empowerment for meeting such goals. This explains the constant investment that should be made in gender-sensitive policies [31,46,52,58,64].

## Conclusions

This study used theory and frameworks related to policy, food systems and gender to understand the extent to which gender is considered in policy – and in particular food system policy related to fruits and vegetables – in four low- and middle-income countries. We find that all study countries have ratified several conventions and international declarations that promote gender equality and have set up policies, strategies and programs aiming to address gender issues explicitly. However, the degree of alignment between these commitments and national food system policies varies. Many policies reviewed in the study countries are gender-blind, failing to recognize the cultural and social norms that often limit women’s access to resources and opportunities in food systems. Women continue to face systemic discrimination within food systems. This discrepancy presents a critical area for improving policy design and implementation. Among other responses, sex-disaggregated data are vital for evidence-based policymaking in the food system; however, very few policy documents have explicitly considered sex-disaggregated data in the study countries. Sex-disaggregated data is important in formulating food system policies that work because it brings out the specific roles and challenges of women and men in the food system, allowing interventions that can be specifically designed to improve dietary diversity for healthy diet. Nevertheless, governments will need to back up a strong policy with strong implementation and enforcement if this is to truly operationalize gender equity in food systems.
